# Brain regions essential for improved lexical access in an aged aphasic patient: a case report

**DOI:** 10.1186/1471-2377-6-28

**Published:** 2006-08-17

**Authors:** Marcus Meinzer, Tobias Flaisch, Jonas Obleser, Ramin Assadollahi, Daniela Djundja, Gabriela Barthel, Brigitte Rockstroh

**Affiliations:** 1 University of Konstanz, Department of Psychology, Universitätsstr.10, P.O. Box 23, 78457 Konstanz, Germany; 2 University College London, Institute of Cognitive Neuroscience, 17 Queen Square, WC1N 3AR, London, Great Britain

## Abstract

**Background:**

The relationship between functional recovery after brain injury and concomitant neuroplastic changes is emphasized in recent research. In the present study we aimed to delineate brain regions essential for language performance in aphasia using functional magnetic resonance imaging and acquisition in a temporal sparse sampling procedure, which allows monitoring of overt verbal responses during scanning.

**Case presentation:**

An 80-year old patient with chronic aphasia (2 years post-onset) was investigated before and after intensive language training using an overt picture naming task. Differential brain activation in the right inferior frontal gyrus for correct word retrieval and errors was found. Improved language performance following therapy was mirrored by increased fronto-thalamic activation while stability in more general measures of attention/concentration and working memory was assured. Three healthy age-matched control subjects did not show behavioral changes or increased activation when tested repeatedly within the same 2-week time interval.

**Conclusion:**

The results bear significance in that the changes in brain activation reported can unequivocally be attributed to the short-term training program and a language domain-specific plasticity process. Moreover, it further challenges the claim of a limited recovery potential in chronic aphasia, even at very old age. Delineation of brain regions essential for performance on a single case basis might have major implications for treatment using transcranial magnetic stimulation.

## Background

Aphasia, the loss of the ability to produce or comprehend language, constitutes a frequent consequence of left hemispheric stroke. Within the aphasic syndrome, one of the most frequent and disturbing syndromes is impaired lexical access (e.g. object naming). Even though patients may succeed in retrieving words, semantic paraphasias (misnaming) and neologisms (meaningless word creations) are frequently observed. Neurophysiological changes accompanying recovery of function in aphasia have remained controversial [1,2] Even though recently two studies used functional magnetic resonance imaging (fMRI) to investigate overt language production in aphasia [3,4] the neural substrate for lexical access and its different types of errors has never been investigated. Furthermore, recent efforts in aphasia treatment [5,6] addressed naming performance by fascilitation or inhibition of brain regions using repetive transcranial magnetic stimulation (rTMS). Therefore treatment may be improved if brain regions involved in successful and failing word retrieval can be distinguished.

In the present study, brain activation during word retrieval was examined by functional magnetic resonance imaging in an 80 year old female patient with with chronic aphasia (see methods). An overt-picture naming task was designed and a temporal sparse-sampling procedure [7] realized to assess the activity pattern correlated with correct responses and different error types. Such a design allows to monitor the overt verbal response during an off-phase of the scanner, while the hemodynamic response is acquired after a short time delay. Furthermore, post-hoc categorisation of different responses types (e.g. correct word retrieval, semantic paraphasias, neologisms) is permitted. In the present study patterns of brain activation were compared for correct responses and errors. Moreover, if there was a difference of activation for errors and correct word retrieval, this should be replicable by comparing a patient's erroneous responses before therapeutic intervention to responses to the same items named correctly after therapy.

### Case presentation

**Figure 1:**
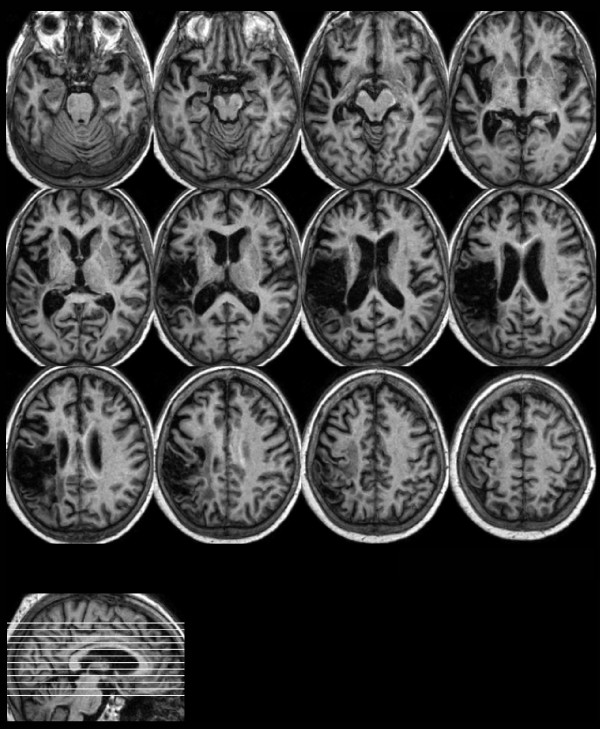
The patient's lesion: The figure shows the normalised T1-weighted structural magnetic resonance scan. The left hemispheric lesion includes the inferior parietal lobe, the superior temporal lobe, postcentral regions, the rolandic operculum, parts of the insula and the inferior frontal gyrus (axial slices, left = left).

We investigated an 80-year-old female patient with chronic Wernicke's aphasia (duration >2 years) following left hemispheric ischemic stroke (Fig. 1 shows the patient's lesion) of the middle cerebral artery who demonstrated a severe word retrieval deficit. The patient was tested before (T1) and after (T2) short-term intensive language training (Constraint-Induced Aphasia Therapy, CIAT [8,9]), which comprises communicative language-games with a focus on word retrieval and is scheduled for three hours/day on ten consecutive days. Three healthy right-handed female controls (aged 76, 80, 85 years) underwent the same fMRI-paradigm twice within two weeks.

## Methods

Language performance of the patient outside of the scanner was evaluated by a standardised language test (Aachen Aphasia Test, AAT [10]) and a naming test that included 150 items. Responses during the naming test were required without time restriction, while the first response of the patient was scored. To account for the potential improvement of working-memory functions and more general attentional processes after therapy two non-language tasks were administered: Visual working-memory was evaluated with the Benton Visual Retention Test [11]. More general attentional processes were assessed by a german non-verbal test of attention and concentration (Alterskonzentrationstest, AKT [12]), which constitutes an adaption of the D2 Test [13] and is specifically tailored to assess attentional capacities of the elderly or aphasic subjects.

MRI was performed on a 1.5 Tesla scanner (Philips Intera) at the Kliniken Schmieder in Allensbach. Functional MRI stimulation consisted of two alternating blocked conditions (overt picture naming/passively viewing a fixation cross; 5 consecutive stimuli for each condition, the sequence of picture-naming blocks was fixed at both sessions while picture presentation during trials was randomized). Pictures were taken from an internet database [14] and included 80 line drawings of objects, high name agreement scores for all stimuli was assured (average name agreement: 0.92 ± 0.09). Forty of these pictures were included in the therapy material and trained once daily. Stimuli were presented by a visor (VisuaStim, Resonance Technology, Inc.) for 3 seconds during which the overt verbal response had to be performed (responses were transmitted by a microphone, tape recorded and transcribed after scanning). After a short delay (0.8 sec.) a whole-brain functional MR volume was acquired (TR = 6 sec.; acquisition time TA = 2.2 sec.; 28 transversal slices, slice-thickness: 4.5 mm; resolution: 3.6 × 3.6 mm, Field of View = 230, acquisition matrix 64 × 64). Data was modeled using a finite impulse response function. A total of 160 functional whole brain volumes were acquired. No overt response was required for the control condition (fixation).

Functional MRI post-processsing was performed using Statistical Parametric Mapping (SPM2, Wellcome Department of Cognitive Neurology, London, UK) and included standard slice-timing, realignment, normalization and smoothing (9 × 9 × 12 mm^3 ^Gaussian Kernel) routines. Normalization in the patient included cost-function masking to prevent distortion of the image due to the lesion [15]. The design allowed post-hoc categorization of the patient's responses as correct naming, semantic paraphasia or neologism, rendering it effectively into an event-related design due to the temporally sparse imaging applied. To elucidate the differential neural contribution to correct word retrieval and error types a total of 26 correct responses, semantic paraphasias and neologisms were chosen from both sessions (13 from each session, response types were matched for length, frequency, naming agreement, visual complexity within and between response types). To assess the general activation pattern of correct responses and error types we calculated basic contrasts comparing correct responses and error types to rest (correct>fixation, semantic paraphasias>fixation, neologisms>fixation). To assess the differential neural substrate of correct responses and error types contrasts included correct responses versus semantic paraphasias (Correct>SP) and correct responses versus neologisms (Correct>Neo). Since the patient's performance improved after therapy we compared activation elicited by 10 selected trials that were categorized as errors (7 neologisms, 3 semantic paraphasias) before therapy to activation elicited by the same pictures after therapy, when the patient articulated a correct response. The general activation pattern elicited by naming in the control group was calculated as a fixed effects model (naming>fixation at T1). To assess changes of activation between sessions in the control group we compared naming before and after the two week time interval (T2>T1). Maximally activated voxels within significant clusters are reported. Anatomic localization was conducted using the Talairach Demon software [16].

Written informed-consent has been obtained from all participants. The study was approved by the local ethics committee and in accordance with the Helsinki Declaration.

## Results

**Figure 2:**
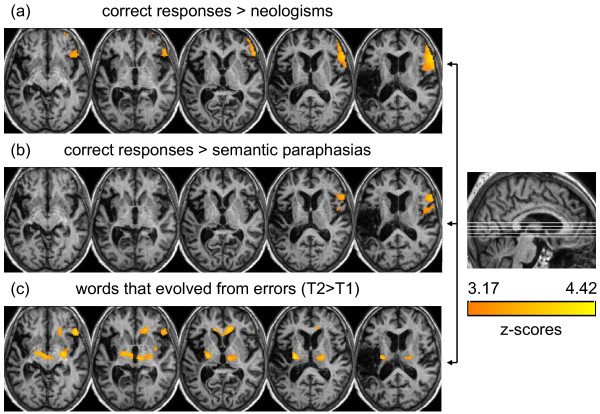
Differential activation for correct and erroneous responses: Increased activation in the right IFG was found comparing correct word retrieval to (a) neologisms and (b) semantic paraphasias. (c) Correct word retrieval at T2, but not at T1 (the same pictures, N = 10) yielded increased activation in the right IFG, the right thalamus and right and left putamen (left = left, all contrasts: cluster threshold p < .05, k>50, FWE-corrected; voxel threshold p < .01, uncorrected).

We compared brain activation elicited by a total of 26 correct naming responses during the overt picture naming task to 26 semantic paraphasias and 26 neologisms. Comparison of correct responses and error types during fMRI yielded a differential activation pattern that was mainly characterised by increased activation in the right inferior frontal gyrus (IFG) for correct words compared to paraphasias and neologisms (Figure 2a+b; Tab. 1, cluster threshold p < .05 corrected, k>50; voxel threshold p < .001, uncorrected; the general activation pattern for correct responses, paraphasias and neologisms is described in Tab. 2). Most interestingly, the difference between the activation pattern of correct responses and error types was more pronounced in the right IFG when responses were semantically less related to the target picture (Fig. 2a+b: words > semantic paraphasias > neologisms).

After a two week period of intensive language training [8] the patient improved significantly in the profile score of a standardised language test (Aachen Aphasia Test, AAT [10]), which is an index of the general aphasia severity, and the repeating subtest of the AAT. Naming performance during fMRI (pre/post: 13/23 correct responses; maximum 80) and the naming test outside of the scanner (pre/post 22/44; maximum 150) almost doubled. No improvement of visual working memory or attentional functions were observed (Tab. 3). Improvement of language performance was mirrored by changed brain activation in the fMRI naming task: when we compared correct responses after therapy that evolved from errors (thus, the same pictures before and after treatment; T1/T2) significantly increased activation was evident in the IFG, the right thalamus, right and left putamen and the anterior cingulate gyrus (cluster threshold p < .05 corrected, k>50; voxel threshold p < .01, uncorrected; Fig. 2c). Naming performance for controls was close to a ceiling effect at both sessions (mean T1/T2: 71/72 correct responses, Tab. 3). The fMRI activation pattern for naming compared to rest (fixation) in controls was comparable to that described in earlier studies using covert or overt picture naming tasks (for review [17]). Picture naming activated a large bilateral network that included frontal, temporal and occipital regions, the thalamus, basal ganglia and limbic structures (Tab. 4). The strongest change of activation in the control group was a decrease of activation in the left IFG at T2 (cluster threshold p < .01 corrected, k>50; voxel threshold p < .001), no increased activation was evident even at a lower threshold (voxel threshold p < .01).

## Discussion

This case-study constitutes the first report to elucidate the neural substrate of correct word retrieval and error performance in aphasia. Differences between correct retrieval and errors were observed mainly in right inferior frontal areas. This was replicated after therapy: words correctly produced at T2, but erroneous at T1, exhibited increased activation in the right IFG and subcortical structures (right thalamus, right and left putamen). The thalamus seems crucially involved in semantic-lexical retrieval by mediating activity in frontal areas [18]. In the present case, this is supported by the gradually increasing involvement of IFG with increasing semantical relationship of responses to the target picture. Furthermore, effective compensatory activity of the right hemispheric IFG homologue during naming performance may depend on intact function of the left basal ganglia [6]. This would explain the increased activation in the right and left putamen that was associated with correct word retrieval after therapy.

The contribution of the left or right hemisphere to the recovery of language in aphasic patients following stroke is controversially discussed in the literature, in particular, activity in right hemispheric areas following stroke has been attributed to "...the loss of active inhibition or competitive interaction of the homologous (left frontal) area, or an inefficient dead-end strategy" [6,19]. Several functional imaging studies on naming in aphasic patients showed no correlation between right hemispheric (frontal) activation and speech recovery [20-23], on the other hand, there are reports that emphasize the importance of right hemispheric areas to the recovery of function in aphasia [24]. For example, Winhuisen et al. [25,26] showed that deactivation of the right IFG using rTMS interferred with performance during a semantic task in aphasic patients, indicating an essential contribution of this area to language recovery (at least in a subset of patients) and results of Crosson et al. [27] support the importance of right frontal areas in successful naming therapy.

In the present study we found gradually increased activation of right the IFG to be associated with better naming performance, which emphasizes the importance of this area for the recovery of naming performance in this particular patient. Still, language performance of the patient was low and even though improvements were substantiated, the overall naming capacity was still severely disturbed after therapy, which in some way points to a dysfunctional compensatory activation pattern. We can only speculate about the effect of rTMS to the right IFG in this patient and it might even be possible that inhibition of this area fascilitates naming performance. On the other hand, the results of the present case study show, that gradual involvement of the right IFG contributes to improved language functions, which makes the previous assumption unlikely.

We also substantiated additionally activated clusters in left perilesional (precentral) areas for correct responses compared to neologisms and sematic paraphasias (see Tab. 1), that may be functionally as important as the right hemispheric activation. However, in patients with primary progresssive aphasia [28] activation in precentral areas was negatively correlated with performance during a naming task using fMRI. The authors interpreted these results as "a compensatory spread of language-related neural activity or a failure to suppress activity in areas normally inhibited during language tasks". While to our knowledge no studies found associations between precentral activation and semantic tasks, activation of the right IFG has been implicated with semantic decisions by a number of studies [25,26], which emphasizes its potential importance during recovery of naming perfomance in aphasic patients. Moreover, left precentral areas were not activated when we compared correct trials vs. former errors, which provides additional evidence for the importance of the right hemispheric IFG for improved task performance in this particular patient, rather than that of the left precentral activation pattern.

One critical issue in this case report is the relatively small number of improved responses during fMRI post-testing compared to the baseline. Still, naming performance was elicited under rigorous time constraints. Naming of the pictures had to be performed within three seconds. This might be an explanation for the relatively low number of improved namings. Moreover, taking into account the relatively low number of correctly named pictures at the first investigation (13/80) an increase by 10 correct namings equals an improvement of 77%. Furthermore, not only the number of correctly namind pictures increased, moreover, the number of neologisms halved (28/14) and the number of semantic paraphasias increased (i.e. there were more semantically related namings than before treatment). Therefore a closer look at the behavioral data during fMRI (obtained under rigorous time constraints) reveals an overall increase of semantically improved naming attempts after treatment. An additional naming test outside of the scanner without time restrictions was performed before and after treatment that included only untrained items. We also found an increase of correct naming responses of 83% (per/post: 24/44), when we also scored self-corrections, this increase was even more pronounced (31/63). Therefore, we are confident that there was an actual improvement of naming performance due to the treatment. This cannot merely be attributed to the training of specific items, rather the generalization to untrained items points to a general fascilitation of lexical access.

Some methodological issues need to be discussed: First, the most crital analysis (correct trials vs. former errors; Tab. 1) was based on 10 items, which is a comaparably week data basis. However, it has been shown convincingly that especially in temporal sparse sampling fewer trials are necessary to achieve the same power in brain activation [7]. With respect to that, we were optimistic to achieve a reliable activation pattern even with 10 trials per condition. In auditory fMRI, a recent study [29] replicated the now well-established finding of left anterior STS activation with intelligible over unintelligble speech material [30-32] on the basis of a mere 10 trials per condition. Therefore, we feel that it was both justified and conclusive to use even a comparably sparse data basis in performing this revealing contrast of error trials which have been corrected in post-testing.

Second, aphasic patients generally fluctuate in their language performance and multiple baseline investigations might be considered essential for the interpretation of therapy induced effects. In the present case report we did not assess the patient's performance during multiple baseline sessions for several reasons: First of all, the patient was in the late chronic stage of aphasia and major spontaneous improvement of language functions within short time intervals are not to be expected [33]. This would be different for the acute or post-acute stage, where spontaneous recovery and stronger fluctuations of behavioral performance would call for multiple baselines in any case.

Furthermore, there is evidence that repeated scanning produces stable activation patterns, even in severe chronic aphasia [34], in this study repeated scanning yielded a decrement of activation, supposedly related to task familiarity, rather than an increase of activation. Therefore, the increase of activation found in the present report is unlikely to be interpreted as the effect of the repeated measurement. From a more pragmatic viewpoint we chose not to asses stability in this 80-year old patient for the following reasons: Since we used the same pictures for pre-post assessment, repeated measures would have familiarized the patient with the test materials and an additional fMRI scan would have increased the stress upon the patient, this was especially important in this 80-year old lady. Furthermore, we included a gender and age-matched control group that evidenced no increased activation. This shows stability of the activation pattern induced by the paradigm across time.

## Conclusion

We conclude that improvement in various language tasks is supported by improved communication within a larger functional cortical network that includes right hemispheric fronto-thalamic areas. This communication might be mediated by spared left basal ganglia functions. Moreover, the crucial impact of right hemispheric (frontal) areas for correct word retrieval evident in the present case supports the potential role of contralateral homologue brain areas in the recovery from aphasia [35].

The findings reported gain significance insofar as they reflect functional changes that are specific both to treatment and to the language domain: First, age-matched controls did not show changes in performance or increased brain activation, which allows to link changes observed in the patient to the treatment supplied. Second, the patient did not improve in less domain-specific measures of brain functioning: performance in both non-language specific tests did not improve after therapy. This ties the changes in brain activation even closer to therapy-induced recovery of language performance. Still, evidence suggests that there is considerable variablility in areas responsible for recovery of language functions in aphasia and the results of the present study are based on a single patient. Therefore, the findings from this case report might not generalize and replication in a larger sample is necessary.

Although based on data of a single patient, the global behavioral improvement after short-term intensive treatment [8,9] challenges the claim of a limited recovery potential in chronic aphasia, even at very old age. This encourages further study of both therapy effectiveness and brain plasticity in aphasia-therapy, where elder patients form the rule rather than an exception. Furthermore, delineation of brain regions essential for language performance in aphasia on a single case basis, might be of major relevance to future treatment efforts using rTMS [5,6].

## Competing interests

The author(s) declare that they don't have any competing interests.

## Authors' contributions

MM conceived and conducted the study, analysed the data and drafted the manuscript. TF, JO and RA were involved in the implementation of the fMRI design and set-up. TF and JO also provided their expertise in data analysis. DD and GB were responsible for diagnostic assessment and conducted therapy. BR, TF and JO participated in the discussion and final revision of the manuscript. MM, TF and JO revised the initial submission. All authors read and approved the final version of the manuscript.

## Pre-publication history

The pre-publication history for this paper can be accessed here:



## Figures and Tables

**Table 1:** Differential brain activation of words and errors: Activation elicited by correct word-retrieval was compared to semantic paraphasias (words > semantic errors) and neologisms (words > neologisms). Furthermore, we compared activation elicited by erroneous responses (before therapy) to the same stimuli that were named correctly after therapy (correct trials > former errors; all contrasts: cluster threshold p < .05, k>50, FWE-corrected; voxel threshold p < .01, uncorrected).

	Hemi	Regions	BA	Z	k	x	y	z
*Words > Neologisms*	R	Inferior frontal	47	4.74	1635	42	28	**-19**
			45	4.51		59	19	**21**
		Middle frontal	46	4.19		50	42	**20**
	L	Frontal (precentral)	6	4.01	499	-50	-3	**47**
	L	Superior frontal	9	4.00	466	-21	43	**39**
*Words > Semantic Errors*	L	Frontal (precentral)	4	3.81	995	-50	-4	**44**
	R	Inferior frontal	45	3.78	629	59	18	**18**
*Correct Trials > Former Errors ****(T2> T1)***	R	Putamen		4.31	562	30	-14	**9**
	L	Putamen		4.24		27	-9	**-7**
	R	Thalamus		3.94		9	-17	**1**
	**R**	Inferior frontal*	**47**	**4.11**	**437**	**42**	**28**	**-17**
		**Middle frontal**	**11**	**4.08**		**27**	**37**	**-17**
		**Limbic (anterior cingulate)**	**24**	**3.92**		**9**	**32**	**1**

Hemi = Hemisphere; R = right, L = left; BA = Brodman Area; k = cluster extent; x/y/z = coordinates according to Talairach and Tournoux [36], Z = z-scores of significant voxels within significant clusters are reported (p < .01, k>50, corrected).

* The right IFG activation for the contrast correct trials > former errors (42,28,-17) differs with regard to localization to that from the contrast words vs. semantic errors, while the localization of the IFG related activation of this contrast and the contrast words vs. neologisms almost match perfectly. This might be explained by the fact that the first contrast includes 7 neologisms (and 3 sematic paraphasias) that may "dominate" the activation pattern.

**Table 2:** General activation pattern of the patient during picture naming for different response types. Contrasts included correct responses > fixation; semantic paraphasias > fixation; neologisms > fixation (cluster threshold p < .01, k>50, FWE-corrected; voxel threshold p < .001, uncorrected).

			**Correct > Fixation**				**SP > Fixation**				**Neo > Fixation**			
**Region**	**Hemi**	**BA**	**x**	**y**	**z**	**Z**	**x**	**y**	**z**	**Z**	**x**	**y**	**z**	**Z**

**OCCIPITAL**														

**Middle Occipital**	R	19	33	-89	21	>9	36	-86	21	>8	36	-86	21	>11
	L	19					-39	-78	12	4.4				
	R	18	24	-95	19	>7								
	L	18	-18	-98	19	5.3								
**Inferior Occipital**	L	19					-39	-79	-1	4.8				
**Fusiform Gyrus**	R	18	27	-83	-21	6.3					30	-83	-21	7.6
**Lingual Gyrus**	R	17	-21	-90	-3	5.2								
	L	17					-21	-88	-3	5.7				
**Cuneus**	R	19					27	-86	35	6.7	27	-86	35	>9
**Uncus**	L	36					-24	-4	-28	6.8				

**TEMPORAL**														

**Middle Temporal**	R	19	45	-81	18	>9	45	-81	18	7.7	45	-81	18	>10
	R	21					53	-21	-4	6.0	53	-20	-1	7.6
**Superior Temporal**	R	38					30	19	-36	6.6				
	R	22					48	-35	5	6.2	48	-29	1	6.8
	L	22					-65	-3	0	5.4				

**FRONTAL**														

**Precentral**	R	4	12	-31	71	7.4								
	L	6					-62	1	28	6.6				
**Inferior Frontal**	R	13*	39	27	7	5.1								
	L	47	-24	29	-6	5.7								
	L						-53	38	1	4.2				
**Middle Frontal**	R	10	45	48	20	4.6								
	R	6					36	-6	61	6.6				
	L	11	-27	38	-4	4.9								

**PARIETAL**														

**Postcentral**	R	3	12	-37	68	7.5								
**Inferior Parietal**	L	40									-39	-47	47	4.0
**Superior Parietal**	L	7									-30	-49	61	5.7
**Precuneus**	L	7									-30	-47	49	5.1

**LIMBIC LOBE AND SUBCORTICAL STRUCTURES**														

**Limbic (Uncus)**	R	38	-27	4	-33	4.5								
	R	34					12	-1	-25	6.2				
	L	36	-21	-2	-30	4.8					-24	-4	-28	6.9
	L	20	-30	-19	-29	4.9								
**Para-hippocal**	L	36					-30	-28	-26	5.6				
**Thalamus**	R						12	-15	1	5.7				
**Globus Pallidus**	R		24	-9	0	6.9								
**Putamen**	L		-15	11	-11	3.5								

SP = semantic paraphasias, Neo = neologisms; Hemi = Hemisphere; R = right, L = left; BA = Brodman Area; x/y/z = coordinates according to Talairach and Tournoux [36], Z = z-scores of significant voxels within significant clusters are reported (p < .001, k>50, corrected).

* The strongest activation in the cluster that includes the right IFG (total cluster size: k = 218, number of voxels located in the right IFG: k = 148) was found in BA 13 (k = 31), closer inspection of this cluster revealed addional activation in BA 45 (29,26,4; Z = 5.0; k = 33), BA 46 (42, 30, 10; Z = 4.8; k = 38) and BA 47 (42,26,1; Z = 4.4, k = 46). Therefore, a substantial part of Brodmann's Areas 45/47 are activated above baseline. Additionally activated voxels were located in the insula (k = 22), the middle frontal gyrus (k = 45) and the superior temporal gyrus (k = 3).

**Table 3:** Test performance of the patient and 3 healthy age-matched female controls. The patient was investigated before and after intensive language therapy. For controls fMRI-scanning was repeated witin a 2-week interval.

**Patient **(80 years, 25 months post stroke)					
Aachen Aphasia Test (* = significant according to AAT manual)					

	**before therapy**			**after therapy**	
**AAT profile score**	47.5			48.9*	
**AAT Token Test (errors)**	27			28	
**AAT repeating**	88			108*	
**AAT written language**	38			39	
**AAT Naming**	44			51	
**AAT comprehension**	90			88	

***Naming Test (no time limit)***					

***150 items (all untrained)***	**before therapy**			**after therapy**	
**correct responses**	24			44	
+ correct after self-correction	+7			+19	

***fMRI-Naming *** *(before/after therapy)*					

***80 items (40 trained)***	**correct**	**SP**	**PP**	**Neo**	**no response**
**trained items (40)**	8/14	11/12	3/7	10/6	8/1
**untrained items (40)**	5/9	11/16	4/3	18/8	2/4
**total**	13/23	22/28	7/10	28/14	10/5

***Benton Visual Retention Test***					

***15 items***	**before therapy**			**after therapy**	
**correct recognition**	11/15			9/15	

***Alterskonzentrationstest (AKT)***					

	**before therapy**			**after therapy**	
**time to completion**	79 s			101 s	
**correct responses (max.20)**	20			19	

**Healthy female controls (N = 3, all ♀)**					
***fMRI Naming (80 items)***					

**ID**	**age**	**correct at T1**		**correct at T2**	
**1**	76	74 (92.5%)		75 (93.75%)	
**2**	80	68 (85%)		69 (86.25%)	
**3**	85	70 (87.5%)		71 (88.75%)	

SP = semantic paraphasia, PP = phonematic paraphasia, Neo = neologism

**Table 4:** General activation pattern of the control group comparing naming with baseline activation (naming > fixation) at the first investigation (fixed effects model, N = 3; cluster threshold p < .01, k>50, FWE-corrected; voxel threshold p < .05 corrected).

**Region**	**Hemisphere**	**BA**	**k**	**x**	**y**	**z**	**Z**
**Frontal (precentral)**	Left	6	6112	-53	-3	26	>13
	Left	4		-50	-10	41	>9
	Left	44		-55	10	5	>8
**Middle Occipital**	Right	18	11466	22	-91	14	>13
	Left	18		-22	-97	10	>12
**Parietal (precentral)**	Right	4	4597	55	-5	17	>12
**Frontal (precentral)**	Right	6		46	-8	28	>11
**Middle Temporal Gyrus**	Right			61	-27	1	>7
**Inferior Frontal Gyrus**	Left	47	373	-30	27	-10	7.09
	Left	47		-24	15	-11	5.91
**Parietal (Precuneus)**	Left	7	513	-22	-68	38	6.91
**Occipital (Precuneus)**	Left	31		-26	-67	24	5.69
**Superior Temporal Gyrus**	Left	41	110	-40	-31	3	6.23
**Middle Frontal Gyrus**	Right	11	111	30	36	-15	6.19
**Limbic Lobe (anterior cingulate)**	Left	32	59	-16	37	4	5.53
**Medial Frontal Gyrus**	Right	6	247	2	-5	61	5.42
**Superior Frontal Gyrus**	Right	6		8	1	68	5.37
	Right	6		18	-6	68	5.33
**Thalamus (Medial Dorsal Nucleus)**	Right		107	8	-15	6	5.30
**Basal Ganglia (Putamen)**	Left		77	-28	-18	-8	5.14

Hemi = Hemisphere; R = right, L = left; BA = Brodman Area; k = cluster extent; x/y/z = coordinates according to Talairach and Tournoux [36], Z = z-scores of significant voxels within significant clusters are reported (p < .05, k>50, FWE-corrected).
